# Direct Comparisons of 2D and 3D Dental Microwear Proxies in Extant Herbivorous and Carnivorous Mammals

**DOI:** 10.1371/journal.pone.0071428

**Published:** 2013-08-06

**Authors:** Larisa R. G. DeSantis, Jessica R. Scott, Blaine W. Schubert, Shelly L. Donohue, Brian M. McCray, Courtney A. Van Stolk, Amanda A. Winburn, Michael A. Greshko, Mackie C. O’Hara

**Affiliations:** 1 Department of Earth and Environmental Sciences, Vanderbilt University, Nashville, Tennessee, United States of America; 2 Department of Sociology and Anthropology, University of Arkansas at Little Rock, Little Rock, Arkansas, United States of America; 3 Center of Excellence in Paleontology and Department of Geosciences, East Tennessee State University, Johnson City, Tennessee, United States of America; 4 Department of Anthropology, Vanderbilt University, Nashville, Tennessee, United States of America; University of Arkansas, United States of America

## Abstract

The analysis of dental microwear is commonly used by paleontologists and anthropologists to clarify the diets of extinct species, including herbivorous and carnivorous mammals. Currently, there are numerous methods employed to quantify dental microwear, varying in the types of microscopes used, magnifications, and the characterization of wear in both two dimensions and three dimensions. Results from dental microwear studies utilizing different methods are not directly comparable and human quantification of wear features (e.g., pits and scratches) introduces interobserver error, with higher error being produced by less experienced individuals. Dental microwear texture analysis (DMTA), which analyzes microwear features in three dimensions, alleviates some of the problems surrounding two-dimensional microwear methods by reducing observer bias. Here, we assess the accuracy and comparability within and between 2D and 3D dental microwear analyses in herbivorous and carnivorous mammals at the same magnification. Specifically, we compare observer-generated 2D microwear data from photosimulations of the identical scanned areas of DMTA in extant African bovids and carnivorans using a scanning white light confocal microscope at 100x magnification. Using this magnification, dental microwear features quantified in 2D were able to separate grazing and frugivorous bovids using scratch frequency; however, DMTA variables were better able to discriminate between disparate dietary niches in both carnivorous and herbivorous mammals. Further, results demonstrate significant interobserver differences in 2D microwear data, with the microwear index remaining the least variable between experienced observers, consistent with prior research. Overall, our results highlight the importance of reducing observer error and analyzing dental microwear in three dimensions in order to consistently interpret diets accurately.

## Introduction

Dental microwear, the microscopic wear patterns resulting from food processing, is among the most frequently used and effective proxies to infer diet in extant and extinct animals, including humans and their ancestors. As dental microwear records food consumption during the last few days to weeks of an animal’s life, it can be used to clarify ancient diets and assess dietary responses to changing climates and environments. While microwear has been commonly used by anthropologists and paleontologists since the late 1970s (e.g., [1–3]), the methodologies used to quantify tooth surfaces are highly variable and still debated among researchers, and results generated between and within methods are not directly comparable [4–7].

The pioneering microwear studies of the 1970s and 1980s used scanning electron microscopy (SEM) to document the correlation between size, shape, and orientation of wear features and dietary habits of extant taxa (e.g., [1,2]). These studies standardized methods related to data collection including the type of wear facet analyzed [8], analysis of homologous facets across studied taxa [3,9], specimen coating material and thickness, and beam settings of individual SEM machines [10]. However, the analysis and subsequent interpretation of microwear features assessed via SEM relies on observers counting individual pits and scratches from two-dimensional SEM micrographs [8]. Typically, in herbivorous taxa, a high incidence of scratches relative to pits is interpreted to indicate the consumption of tougher food items, potentially with higher silica or grit content; in contrast, a greater frequency of pits indicates the consumption of more brittle objects and the potential processing of seeds and/or fruit pits [1,2,8].

Microwear studies utilizing low-magnification light microscopy follow similar methods as those applied during SEM analysis, with the added benefit of being able to analyze a surface quickly with a low-cost stereo light microscope at magnifications ranging from 35x in large animals (e.g., [11]) to 100x in small animals (e.g., [12]). Here, observers can either directly count wear features through the microscope lens [11], or they can take digital photomicrographs of specimens and thus keep a record of their counts by tracing wear features using imaging software [13,14]. Biplots of pits and scratches yield a ‘trophic triangle’ where, for example, herbivorous browsing taxa have a high number of pits and low number of scratches, grazing taxa have a high number of scratches and low number of pits, and frugivores/hard object feeders fall in-between these end members [11,15]. Further, the addition of features including scratch texture, cross scratches, large pits, and gouges can further parse out dietary information [11].

Observer identification and quantification of individual wear features, whether from an SEM micrograph or using light microscopy, is prone to high observer biases, particularly between observers of different experience levels [4,5,7,16]. Grine and co-authors [4] found a 9% error in measurements between observers while Galbany and co-authors [5] found a 6% error in observers with five or more years of experience when quantifying SEM micrographs. Most recently, Mihlbachler and colleagues [7] documented a 45% interobserver error among experienced and inexperienced individuals, which was reduced to 8-12% in experienced individuals after multiple iterations. While experienced observers yielded similarly shaped trophic triangles consistent with prior work [11], inexperienced microwear observers failed to generate a trophic triangle using light microscopy [7]. Further biases can arise from viewing microwear features at different resolutions [17], in addition to data loss from the analysis of three-dimensional microwear features in two dimensions [6,18]

Dental microwear texture analysis (DMTA), the analysis of dental microwear in three dimensions using a scanning white light confocal microscope and scale sensitive fractal analysis, alleviates some of the problems surrounding traditional microwear methods by essentially eliminating observer bias through a quantitative, repeatable method, and by analyzing wear features in three dimensions [18–20]. While some observer biases may still be present (e.g., area of wear facet chosen for analysis by individual observers), DMTA has been shown to distinguish between disparate diets in mammals including bovids [21], carnivorans [22,23], marsupials [24], primates [18,19], and xenarthrans [25]. Specifically, greater values in features such as complexity (i.e., surface roughness at varying scales) occur in taxa consuming brittle food items, such as frugivores and bone consumers, while greater anisotropy (i.e., the orientation of wear features of similar depth) occurs in grazers and carnivores that avoid bone. In contrast, Beatty and Mihlbachler [26] found that low-magnification microwear methods were better able to discriminate between species than DMTA, although neither method discriminated diet well. However, the Beatty and Mihlbachler [26] study was limited in sample size (total of 15 specimens from four species) and could not compare identical surface areas on each specimen due to methodological constraints.

**Figure 1:**
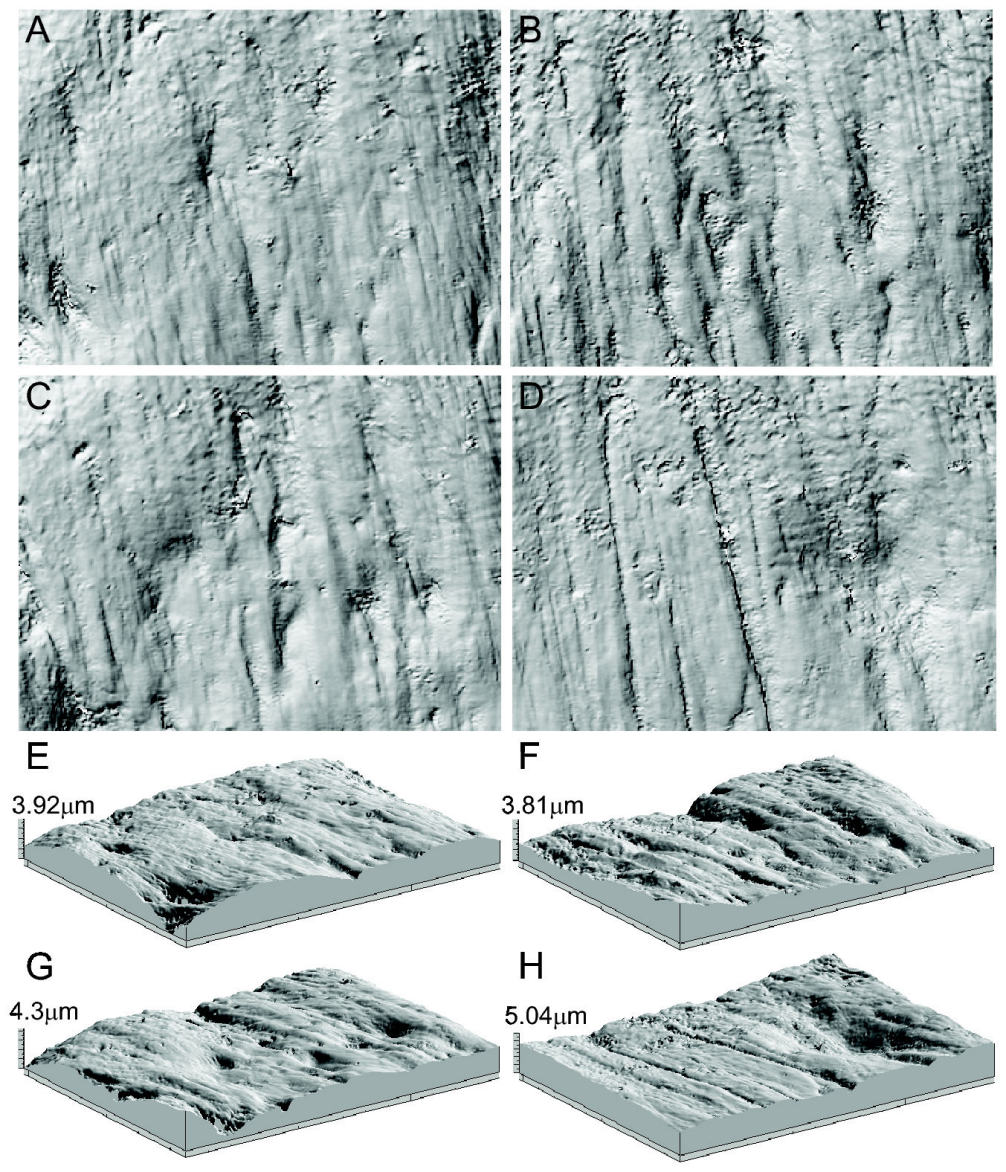
Dental microwear photosimulations in 2D and 3D. Example of photosimulations in two dimensions (A–D) and three dimensions (E–H) of the browser *Sylvicapra* *grimmia* . Scans are from four adjacent areas (totaling ~204 x 276 µm^2^, with each scan representing and area of ~102 x 138µm^2^).

Here, we directly compare 2D and 3D dental microwear features of herbivorous and carnivorous mammals at 100x magnification (Figure 1). Specifically, we compared DMTA data with generated 2D microwear data from photosimulations of the identical scanned areas of previously published DMTA 3D point clouds of extant African bovids (4 species [21]) and carnivorans (3 species [23]). Photosimulations avoid the pitfalls of SEM and stereo light microscopy methods, which can be subject to the issue of depth distorting the visibility of microwear features due to differences in coating thickness, SEM beam angle, or angle of incoming light, among other factors. The view settings for photosimulations can be manipulated to alter the direction of light and photo contrast to maximize feature visibility, while still preserving "observer-perceived depth" via feature shadows. Conducting 2D microwear analysis on DMTA-produced photosimulations is the only method that ensures direct comparisons of 2D and 3D methods between identical scanned areas. Analyzing the identical tooth area using a confocal microscope to generate 3D data and an SEM or stereo light microscope to generate 2D data is virtually impossible and would introduce additional sources of differences. Thus, we analyzed taxa with similar dental morphology (within each group) representing distinct dietary niches, to address the following questions: (i) do two-dimensional and three-dimensional microwear studies produce accurate and comparable dietary interpretations of extant taxa with known feeding behavior, (ii) how does the inclusion of depth alter or improve dental microwear interpretations, and (iii) can different observers generate comparable data using two-dimensional wear feature counting methods in herbivorous and carnivorous mammals? Understanding how 2D and 3D dental microwear data compare in a diversity of mammals with disparate diets is critical to advancing our understanding of dietary behavior in the prehistoric and historic past, globally.

## Results

### Dietary Comparisons

**Figure 2:**
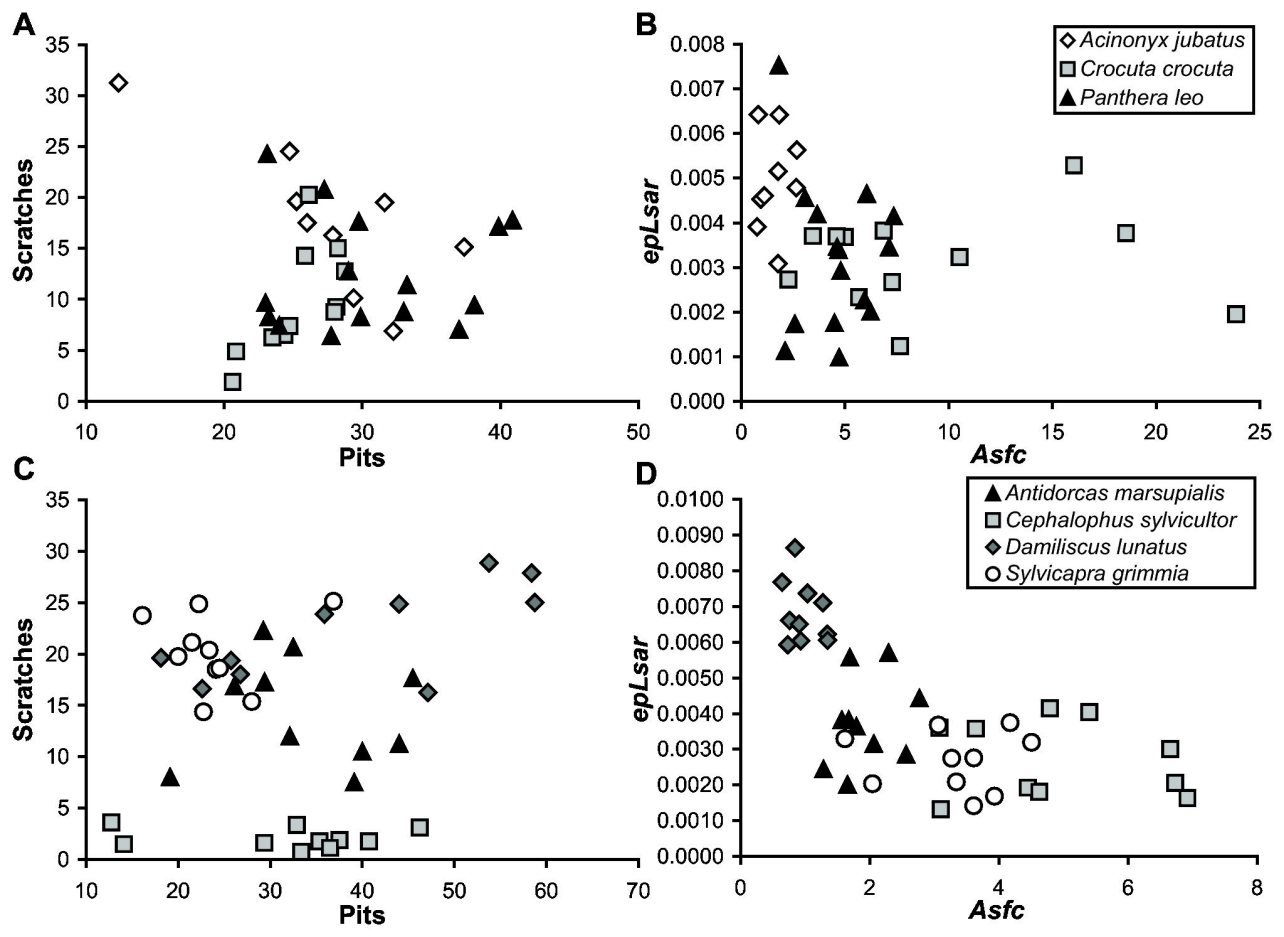
Bivariate plots of dental microwear 2D and 3D data of carnivorans and bovids. Bivariate plots of number of scratches and number of pits from 2D photosimulations of (A) carnivorans, and (C) bovids. Dental microwear texture data (*Asfc*, complexity; *epLsar*, anisotropy) of (B) carnivorans, and (D) bovids, from Refs. 21,23, respectively.

Results are presented in Tables 1-2 and illustrated in Figure 2. All primary data are noted in Tables S1-S4 and the supplemental Appendix Data File. As carnivoran and bovid dietary comparisons are the focus of prior studies [21,23], we instead specify how DMTA data compares to 2D metrics at the same resolution. In the 2D analysis the number of pits and coarse pits are not statistically distinct between carnivorans (Kruskal-Wallis tests, p=0.078 and 0.626, respectively). A greater number of scratches (p=0.017) and coarse scratches (p=0.004) distinguishes *Acinonyx* *jubatus* from *Crocuta* *crocuta* , with *Panthera* *leo* also having statistically greater coarse scratches than *C* *. crocuta* . DMTA attributes instead yield significantly greater parallel elongated wear features (mean anisotropy or *epLsar*) in *A* *. jubatus* than in all extant taxa (Table 1, Kruskal-Wallis tests, p<0.0001). In contrast, mean complexity and textural fill volume are smallest in *A* *. jubatus* , followed by *P. leo*, and *C* *. crocuta* (Table 1), consistent with degree of durophagous activity in these carnivorans (Kruskal-Wallis tests, p<0.01).

**Table 1:** Descriptive statistics for each DMTA variable by species.

**Taxon**	**Statistic**	***n***	***Asfc***	***epLsar***	***Smc***	***Tfv***	***HAsfc*_*(3x3)*_**	***HAsfc*_*(9x9)*_**
*Acinonyx* *jubatus*	Mean	9	1.590	0.0049	0.286	5071	0.589	1.348
(extant carnivoran)	Median		1.767	0.0047	0.209	2581	0.512	1.032
	Standard Deviation		0.737	0.0011	0.154	5372	0.278	0.895
	Skewness (Fisher)		0.424	-0.125	1.12	0.749	0.987	1.464
	*p* for normality (Shapiro-Wilk)		0.165	0.764	0.085	0.147	0.069	0.039
*Panthera* *leo*	Mean	15	4.616	0.0031	1.013	10413	0.471	0.895
(extant carnivoran)	Median		4.690	0.0033	0.150	11358	0.442	0.799
	Standard Deviation		1.729	0.0017	2.596	4074	0.156	0.314
	Skewness (Fisher)		-0.080	0.981	3.566	-0.664	0.67	1.449
	*p* for normality (Shapiro-Wilk)		0.611	0.211	<0.0001	0.042	0.443	0.032
*Crocuta* *crocuta*	Mean	12	9.315	0.0031	0.151	12320	0.462	0.836
(extant carnivoran)	Median		7.070	0.0034	0.151	14142	0.415	0.700
	Standard Deviation		6.708	0.0011	0.001	5666	0.18	0.333
	Skewness (Fisher)		1.215	0.035	0.504	-0.823	0.725	0.802
	*p* for normality (Shapiro-Wilk)		0.046	0.666	0.151	0.326	0.273	0.072
*Damaliscus* *lunatus*	Mean	10	0.984	0.0068	1.355	2027	0.444	0.658
(extant bovid)	Median		0.922	0.0066	1.305	1666	0.451	0.672
	Standard Deviation		0.260	0.0009	0.363	981	0.073	0.072
	Skewness (Fisher)		0.418	1.045	1.149	0.952	-0.144	-0.161
	*p* for normality (Shapiro-Wilk)		0.239	0.187	0.323	0.041	0.966	0.621
*Antidorcas* *marsupialis*	Mean	10	1.937	0.0037	0.366	5709	0.527	0.769
(extant bovid)	Median		1.744	0.0037	0.305	5476	0.492	0.799
	Standard Deviation		0.474	0.0012	0.187	1189	0.149	0.147
	Skewness (Fisher)		0.634	0.421	1.709	1.291	1.082	-0.397
	*p* for normality (Shapiro-Wilk)		0.418	0.618	0.030	0.188	0.361	0.750
*Sylvicapra* *grimmia*	Mean	10	3.318	0.0027	0.681	12622	0.778	0.895
(extant bovid)	Median		3.477	0.0028	0.510	12357	0.611	0.882
	Standard Deviation		0.900	0.0008	0.327	2834	0.354	0.181
	Skewness (Fisher)		-0.840	-0.149	0.987	-0.178	0.674	0.917
	*p* for normality (Shapiro-Wilk)		0.420	0.523	0.001	0.341	0.044	0.198
*Cephalophus* *sylvicultor*	Mean	10	4.940	0.0027	0.257	12997	0.466	0.820
(extant bovid)	Median		4.710	0.0025	0.265	13671	0.466	0.828
	Standard Deviation		1.459	0.0011	0.027	1973	0.050	0.056
	Skewness (Fisher)		0.165	0.138	-0.623	-0.901	0.841	-1.484
	*p* for normality (Shapiro-Wilk)		0.252	0.154	0.470	0.062	0.089	0.055

*n*, number of individuals sampled; *Asfc*, area-scale fractal complexity; *epLsar*, anisotropy; *Smc*, scale of maximum complexity; *Tfv*, textural fill volume; *HAsfc*_(3x3)_, *HAsfc*_(9x9)_ heterogeneity of complexity in a 3x3 and 9x9 grid, respectively. All carnivoran data and all descriptive statistics are taken from Ref. [23]. Bovid descriptive statistics are not published in this form; however, all specimens were previously analyzed in Ref. [21].

**Table 2:** Descriptive statistics for each 2D dental microwear variable by species.

**Taxon**	**Statistic**	***n***	**Pits**	**CP**	**Scratches**	**CS**	**MI**
*Acinonyx* *jubatus*	Mean	9	27.43	4.19	17.61	1.75	0.782
(extant carnivoran)	Median		27.88	3.50	17.25	1.75	0.609
	Standard Deviation		6.93	1.53	7.24	0.70	0.687
	Skewness (Fisher)		2.635	1.811	0.514	-1.010	6.248
	*p* for normality (Shapiro-Wilk)		0.338	0.066	0.918	0.592	0.002
*Panthera* *leo*	Mean	15	30.61	4.18	12.19	1.208	0.414
(extant carnivoran)	Median		29.75	4.75	9.38	1.125	0.344
	Standard Deviation		6.19	1.04	5.64	0.614	0.228
	Skewness (Fisher)		-1.152	-1.182	-0.499	-0.899	3.274
	*p* for normality (Shapiro-Wilk)		0.189	0.054	0.041	0.430	0.007
*Crocuta* *crocuta*	Mean	12	25.23	3.77	9.20	0.625	0.354
(extant carnivoran)	Median		25.31	3.56	7.81	0.313	0.296
	Standard Deviation		2.79	0.82	5.17	0.612	0.183
	Skewness (Fisher)		-0.947	-0.176	0.221	-1.978	1.163
	*p* for normality (Shapiro-Wilk)		0.297	0.025	0.521	0.014	0.209
*Damaliscus* *lunatus*	Mean	10	39.11	3.61	22.04	2.09	0.972
(extant bovid)	Median		39.94	3.00	21.75	2.19	0.774
	Standard Deviation		15.32	2.05	4.63	0.82	0.527
	Skewness (Fisher)		0.017	1.320	0.171	0.132	2.500
	*p* for normality (Shapiro-Wilk)		0.294	0.215	0.317	0.790	<0.001
*Antidorcas* *marsupialis*	Mean	10	33.71	6.84	14.39	0.96	0.654
(extant bovid)	Median		32.31	6.5	14.44	0.88	0.519
	Standard Deviation		8.34	3.35	5.21	0.87	0.368
	Skewness (Fisher)		-0.139	0.352	0.109	1.454	1.004
	*p* for normality (Shapiro-Wilk)		0.786	0.763	0.443	0.143	0.068
*Sylvicapra* *grimmia*	Mean	10	23.95	3.64	20.19	2.58	1.334
(extant bovid)	Median		23.06	3.31	20.06	2.50	1.285
	Standard Deviation		5.49	0.86	3.69	1.40	0.657
	Skewness (Fisher)		1.396	0.469	-0.131	0.051	0.839
	*p* for normality (Shapiro-Wilk)		0.124	0.089	0.601	0.646	0.684
*Cephalophus* *sylvicultor*	Mean	10	31.89	3.09	2.05	0.11	0.204
(extant bovid)	Median		34.38	2.63	1.75	0.06	0.172
	Standard Deviation		10.75	1.63	0.98	0.14	0.134
	Skewness (Fisher)		-0.966	1.250	0.619	0.863	1.824
	*p* for normality (Shapiro-Wilk)		0.126	0.139	0.161	0.019	0.022

*n*, number of individuals sampled; Pits, number of pits of all size categories; CP, number of coarse pits as defined by Ref. [11]; Scratches, number of scratches of all size categories; CS, number of coarse scratches as defined by Ref. [11]; MI, microwear index as defined by the number of scratches/number of pits, Ref. [36]. All 2D dental microwear features are averages of median values taken from four photosimulations per specimen (see Materials and Methods).

Mean number of pits (ANOVA, p=0.025) is statistically distinct between bovids in the 2D study, with the grazer *Damaliscus* *lunatus* having greater pits than the browser *Sylvicapra* *grimmia* . The number of coarse pits is instead greatest in the grazer-browser *Antidorcas* *marsupialis* as compared to the frugivore *Cephalophus* *sylvicultor* (ANOVA, p=0.002). Although pits can distinguish between some extant bovids, these trends are contrary to expectations of greater pits and coarse pits in browsers and frugivores, in comparison to grazers and generalists, respectively. Mean number of scratches is distinct in bovids (ANOVA, p<0.0001). Predictably, fewer scratches occur in the frugivore *C* *. sylvicultor* , as compared to all other extant bovids analyzed (p<0.0001 for all Tukey HSD tests). Further, *A* *. marsupialis* has fewer scratches than *D* *. lunatus* and *S* *. grimmia* (p=0.001 and 0.012, respectively, Tukey HSD tests).

Although scratch frequency can clearly discriminate between frugivores and grazers consistent with observed diets (i.e., grazers having a greater number of scratches), DMTA variables are better able to discriminate between disparate dietary niches (Figure 2). Specifically, the grazer *D* *. lunatus* has greater anisotropy than all other bovids (Fisher LSD and Tukey HSD tests, p<0.0001), while the intermediate grazer-browser *A* *. marsupialis* has greater anisotropy than *C* *. sylvicultor* and *S* *. grimmia* (Fisher LSD tests, p<0.05). Similarly, the frugivore *C* *. sylvicultor* has the greatest complexity (followed by the browser *S* *. grimmia* , mixed feeder *A* *. marsupialis* , and grazer *D* *. lunatus* ), significantly greater than all other bovids sampled (Fisher LSD and Tukey HSD tests, p≤0.001). The browser *S* *. grimmia* also has greater complexity than *A* *. marsupialis* and *D* *. lunatus* (Fisher LSD and Tukey HSD tests, p<0.01), while *D* *. lunatus* has lower complexity than *A* *. marsupialis* (Fisher LSD tests, p<0.05). Similarly, features such as textural fill volume can be used to distinguish between grazing/mixed feeding (i.e., *D* *. lunatus* and *A* *. marsupialis* ) as compared to browsing/frugivory (i.e., *S* *. grimmia* and *C* *. sylvicultor* ; Kruskal-Wallis test, p<0.0001).

While DMTA provides greater resolution between disparate dietary groups in both herbivorous and carnivorous taxa, there is also congruence between DMTA variables and traditional dental microwear variables (Spearman’s rank correlations). Most notably, anisotropy and total number of scratches and coarse scratches are positively correlated (ρ=0.31, p<0.01; ρ=0.30, p<0.01, respectively). The same is true for scale of maximum complexity (ρ=0.38, p<0.001; ρ=0.36, p<0.01, respectively). Further, scratch counts and coarse scratch counts are negatively correlated with complexity (ρ=-0.59, p<0.0001; ρ=-0.49, p<0.0001, respectively) and textural fill volume (ρ=-0.40, p<0.001; ρ=-0.32, p<0.01, respectively). Pit counts and coarse pit counts are not significantly correlated with DMTA characters, with the exception of a negative relationship between total pit count and textural fill volume (ρ=-0.27, p=0.02). Microwear index values are positively correlated with anisotropy (ρ=0.33, p<0.01) and scale of maximum complexity (ρ=0.45, p<0.0001), and negatively correlated with complexity (ρ=-0.55, p<0.0001) and textural fill volume (ρ=-0.33, p<0.01). Although DMTA variables measure different textural attributes, complexity is positively correlated with textural fill volume (ρ=0.71, p<0.0001) and negatively correlated with anisotropy (ρ=-0.51, p<0.0001) and scale of maximum complexity (ρ=-0.59, p<0.0001). Anisotropy is positively correlated with scale of maximum complexity (ρ=0.33, p<0.01) and negatively correlated with textural fill volume (ρ=-0.36, p<0.01), while scale of maximum complexity is negative correlated with textural fill volume (ρ=-0.23, p=0.046). Heterogeneity measurements do not here reveal dietary differences and are only correlated with each other (ρ=0.73, p<0.0001).

### Observer Differences

**Figure 3:**
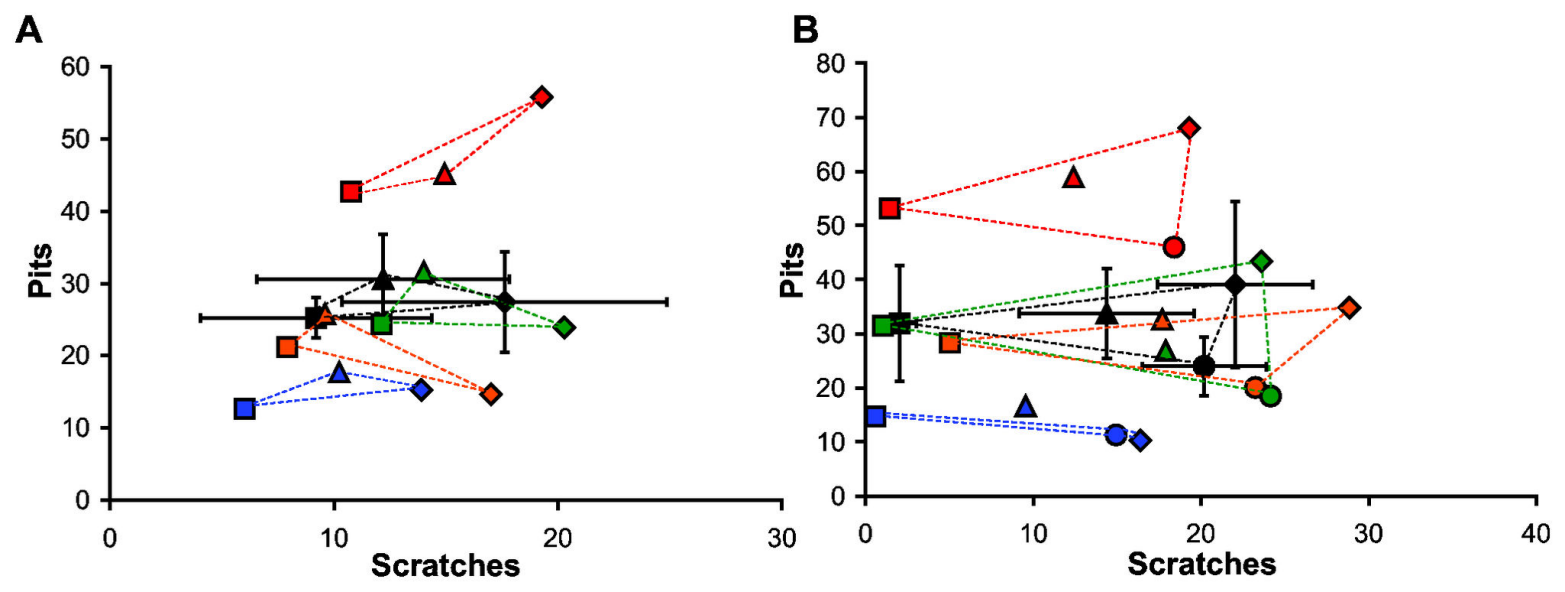
Bivariate plots of number of scratches and number of pits of carnivorans and bovids. Mean values of all four observers +/- 1 standard deviation are noted in black. Individual observer mean values are noted for each observer (1, orange; 2, green; 3, blue; 4, red) for carnivorans (A) and bovids (B). Colored dashed triangles correspond with each individual observer and mean values, connecting all carnivorans (A) and the three dietary categories of grazers, browsers, and frugivores in bovids (B). Carnivoran symbols include: *A* *. jubatus* , diamond; *P*. *leo*, triangle; and, *C* *. crucuta* , square. Bovid symbols include: *D* *. lunatus* , diamond; *A* *. marsupialis* , triangle; *S* *. grimmia* , circle; and, *C* *. sylvicultor* , square.

Results are presented in Tables 3-4 and illustrated in Figure 3. As DMTA attributes are calculated using scale sensitive fractal analysis, microwear data do not vary between observers. In contrast, mean interobserver error of 2D features varies in carnivorans from 30.4% to 463% (mean of 169%) in pit frequency and 12.4% to 98.9% (mean of 60.5%) in scratch frequency. Mean interobserver error for coarse pits and coarse scratches instead range from 33.3% to 166.7% (mean 82%) and from 16.7% to 200% (mean 81.5%), respectively. Bovids instead have significantly lower mean interobserver errors of 49.4% (range of 9.1% to 102%) and 45.4% (range of 6.5% to 150%) in pits and scratches, respectively (p<0.0001, Mann-Whitney tests). Coarse scratches have error percentages of 65.6% (ranging from 0% to 150%), significantly lower (p=0.0002, Mann-Whitney tests) than carnivorans despite the tendency for observers to have highly variable feature counts. Interobserver error of mean coarse pits is 78.2% (ranging from 4.8% to 150%).

**Table 3:** Summary of comparisons using Dunn’s procedure of observer differences between all extant taxa analyzed.

**Dietary Group**	**Taxon**	***n***	**Pits**	**CP**	**Scratches**	**CS**	**MI**
*carnivore, avoids bone processing*	*Acinonyx* *jubatus*	9	p<0.001*	p<0.0001*	p=0.291	p<0.0001*	p=0.014*
*carnivore, generalized degree of durophagy*	*Panthera* *leo*	15	p<0.0001*	p<0.0001*	p=0.068	p<0.0001*	p=0.059
*carnivore, high degree of durophagy*	*Crocuta* *crocuta*	12	p<0.0001*	p<0.0001*	p=0.066	p<0.001*	p=0.068
*herbivore, grazer*	*Damaliscus* *lunatus*	10	p<0.0001*	p<0.0001*	p<0.001*	p<0.001*	p<0.0001*
*herbivore, grazer-browser*	*Antidorcas* *marsupialis*	10	p<0.0001*	p<0.001*	p=0.009*	p<0.001*	p=0.001*
*herbivore, browser*	*Sylvicapra* *grimmia*	10	p<0.0001*	p<0.0001*	p=0.002*	p<0.001*	p<0.001*
*herbivore, frugivore*	*Cephalophus* *sylvicultor*	10	p<0.001*	p<0.001*	p<0.0001*	p=0.004*	p<0.001*

* Significant values (*p*<0.05). *n*, number of individuals sampled; Pits, number of pits of all size categories; CP, number of coarse pits as defined by Ref. [11]; Scratches, number of scratches of all size categories; CS, number of coarse scratches as defined by Ref. [11]; MI, microwear index as defined by the number of scratches/number of pits, Ref. [36]. All 2D dental microwear features analyzed are averages of median values taken from four photosimulations per specimen per observer (see Materials and Methods)

**Table 4:** Paired comparisons of the two most highly trained observers (1 and 2) for all carnivorous and herbivorous taxa analyzed.

**Taxa**	**Statistical Tests**	**Pits**	**CP**	**Scratches**	**CS**	**MI**
**Bovids**	Wilcoxon signed-rank test (mean values, n=40)	p=0.468	p=0.002*	p=0.023*	p=0.256	p=0.900
	Student’s t-test (if appropriate)	p=0.703	N/A	p=0.039*	N/A	N/A
	Wilcoxon signed-rank test (all scans, n=120)	p=0.161	p<0.001*	p<0.010*	p=0.112	p=0.875
**Carnivorans**	Wilcoxon signed-rank test (mean values, n=40)	p=0.001*	p<0.0001*	p<0.0001*	p=0.078	p=0.119
	Student’s t-test (if appropriate)	p=0.001*	N/A	p<0.0001*	N/A	N/A
	Wilcoxon signed-rank test (all scans, n=120)	p<0.0001*	p<0.0001*	p<0.0001*	p=0.029*	p=0.383

* Significant values (*p*<0.05). Pits, number of pits of all size categories; CP, number of coarse pits as defined by Ref. [11]; Scratches, number of scratches of all size categories; CS, number of coarse scratches as defined by Ref. [11]; MI, microwear index as defined by the number of scratches/number of pits, Ref. [36].

Two-dimensional dental microwear data are highly variable, and observer differences are significant in all features of all species, except scratches in carnivorans and microwear index in *P. leo* and *C* *. crocuta* (Table 3). However, when all scans are compared between observers, differences are significant in all but *A* *. jubatus* scratches, which approaches significance (p=0.052; Table S5). While observer differences are apparent, observers maintain consistency in regard to the relative amount of pits and scratches assigned. For example, all observers attribute the greatest mean number of scratches to *A* *. jubatus* , followed by the generalist *P. leo* and scavenger *C* *. crucuta* (Figure 3). In contrast, pit assignments are more variable, although mean pits are consistently greater in *P. leo* as compared to *C* *. crucuta* (Figure 3). Observers are similarly consistent when characterizing scratches, and typically assign the greatest mean number of scratches to either *D* *. lunatus* or *S* *. grimmia* , with *A* *. marsupialis* having more scratches than *C* *. sylvicultor* (Figure 3). Similar to carnivorans, pit assignments are highly variable and not consistent in relative rank order between observers.

Observers 1 and 2 are the most highly trained and have 2D microwear variables most similar to mean values (Figure 3); thus, these observers were compared to one another across all dietary categories. In carnivorans, observer assignments were different in all but coarse scratches and microwear index values (Table 4). In bovids, only coarse pits and scratches were different between observers (Table 4). However, mean interobserver error of observers 1 and 2 is lowest in scratches (24.6%), followed by coarse scratches (26.2%), pits (27.3%), and coarse pits (66.3%) in carnivorans. In bovids, mean interobserver error of observers 1 and 2 is lowest in coarse scratches (25.2%), followed by pits (29.4%), scratches (34.3%), and coarse pits (44.8%).

## Discussion

### Dietary Comparisons

Previous studies of carnivoran 2D microwear differentiate between distinct diets based on foraging behavior [27,28]. Van Valkenburgh and coauthors demonstrated that analysis of scanning electron microscope images of dental microwear (250x) differentiates consumers of large bones such as the spotted hyena ( *Crocuta* *crocuta* ), from other carnivorans, based on the number of pits present on their teeth [28]. Subsequent studies further confirmed these results and suggest that the consumption of large bones can be determined by low resolution (63x) two-dimensional microwear analysis [27]. Specifically, spotted hyenas are statistically distinct from folivores, frugivores, insectivores, malacophages, omnivores, piscivores, and larvae and worm eaters via pit frequency [27]. Contrary to expectations, our two-dimensional microwear results actually demonstrate the absence of statistical distinctions between *C* *. crocuta* and other carnivorans sampled, based on number of pits or coarse pits (Table 2). It is possible that our sampled *C* *. crocuta* differed from expectations based on a difference in diet caused by seasonal or regional variation, or that *C* *. crocuta* has ‘fallback’ resources of bone that are not always utilized [22]. In contrast, our 2D analysis of scratch frequency does differentiate between bone-avoiders and those engaging in greater durophagy, as suggested by prior work [28]. Thus, our results imply that 2D analysis can be successful at distinguishing bone-avoiders, such as *A* *. jubatus* , from bone consumers; however, DMTA analysis of extinct and extant carnivorans demonstrates the ability to infer degree of carcass utilization absent of observer biases and potentially with greater discrimination at the individual specimen level [22,23].

The more extensive literature concerning herbivore microwear suggests that 2D analysis of ungulate molars can distinguish between grazers and browsers, with frugivores and mixed feeders falling in-between [15]. Many studies have sought to characterize past environments based on bovid microwear [29,30]. A comparison of extant and extinct African bovids showed that these herbivores could be reliably identified as browsers or grazers [31]. As many paleoecological studies analyze bovid microwear to assess the presence of open grasslands, being able to identify grazers using microwear is fairly reliable via scratch counts, both here and in prior work [31–33].

Observer-generated 2D microwear features can distinguish extreme dietary categories, such as between frugivores and non-frugivorous bovids, based on number of scratches (Figure 2). Contrary to expectations, we recorded higher numbers of pits for the obligate grazer *D* *. lunatus* than for the browser *S* *. grimmia* . ‘Prism plucking’, wherein small, prism-sized pits are removed from the enamel surface, can result from parallel food processing of tough foods such as grass blades that require shearing or grinding [34,35]. Previous researchers hypothesized that this phenomena caused pits in the teeth of grazers, and thus may have caused the higher number of pits observed for *D* *. lunatus* relative to *S* *. grimmia* . Our findings suggest that coarse designations (e.g., between grazers and frugivores) may be possible using 2D microwear; however, distinguishing between distinct dietary niches in herbivorous taxa such as bovids, requires the inclusion of three-dimensional data. For example, 3D microwear studies have shown that DMTA attributes accurately separated obligate grazers, generalists, browser-grazer intermediates, frugivores, and browsers in extant African bovids [21]. The DMTA analysis of bovids described here derives from the same dataset and demonstrates how increased complexity (*Asfc*) and decreasing anisotropy (*epLsar*) occurs with the predictable consumption of harder and/or less abrasive food items. Lastly, the convergence of similar DMTA results across disparate taxonomic groups (e.g., bovids, macropods, primates, xenarthrans) [18,19,21–25] contributes to greater comparability amongst diverse organisms.

Dental microwear texture analysis attributes record diet in a similar, but more discerning manner, as some traditional dental microwear variables (i.e., scratches and microwear index values). Specifically, photosimulations with greater scratch and coarse scratch frequencies also had higher anisotropy, as expected (i.e., anisotropy is greater when features of similar depth are oriented in similar directions, akin to scratches). In contrast, photosimulations with high numbers of scratches and coarse scratches also had lower complexity and textural fill volume. While anisotropy and complexity are representative of different textural attributes, high anisotropy is usually accompanied with low complexity and vice versa (relationships between these two variables are significant). Number of pits is typically unrelated to most DMTA variables, as individuals consuming hard objects may have fewer but deeper pits while others may have lots of smaller pits; however, individuals with higher textural fill volume (likely containing fewer but larger and/or deeper pits) have significantly fewer pits. As pit counts of like surfaces are highly variable and inconsistent between observers, pits may be harder for observers to discern and less informative. Thus, the use of 3D DMTA data can better differentiate between disparate dietary groups in both herbivorous and carnivorous taxa, likely due to the inclusion of quantitative depth data.

### Observer Comparisons

Overall, highly significant differences were observed between observers in either pits and/or scratches in all taxa. This is consistent with prior methodological studies [4–7,16] and poses a concern for comparing data between observers. Even highly trained observers lack consistency in at least one major feature (i.e., pits or scratches) in both herbivorous and carnivorous mammals (Table 4). Although observer differences in herbivorous taxa are slightly higher than other studies [4–7,16], they are consistent with ranges from these studies when also accounting for the number of observers (which increases the potential for greater observer differences) and when comparing the most highly trained observers. However, as there are no published studies that have calculated interobserver error of microwear features in carnivores, future work is necessary to determine if carnivore microwear counts are consistently highly variable. While observer discrepancies in microwear feature counts raise concern, ratios such as the microwear index may be useful in reducing observer differences in highly trained individuals, as previously suggested [5,7]. For example, an individual who counts more features may have a similar microwear index as another observer if both pit and scratch counts are proportionally inflated.

The rough structure of herbivore 'trophic triangles' is preserved in the most experienced observers and is consistent with prior work [7,15]. While carnivorous 'trophic triangles' are apparent and relatively consistent between the most highly trained observers, pit counts are highly variable and inconsistent (as compared to mean scratches which maintain the same rank order between all observers, Figure 3). Nevertheless, the overall lack of consistency between observers (all mean interobserver error rates are greater than 45%) calls to question the repeatability and comparability of two-dimensional microwear data that is determined by a human observer, particularly in the case of carnivorous mammals (with mean observer errors exceeding 60%).

## Concluding Remarks

Previous studies have indicated that two-dimensional low-resolution microwear analysis provides a fast, cost-effective method for determining gross dietary differences within taxa, such as the difference between grazers and frugivores [11,15], or the difference between bone-avoiders and bone consumers [27]. DMTA, however, is able to distinguish between more subtle dietary niches than two-dimensional high-resolution microwear analysis for both bovids and carnivorans. Further, 2D analyses are highly variable between observers, even those who are highly trained. Collectively, we suggest that quantifying dental microwear in 3D has the potential to distinguish between diverse diets with less variability than 2D methods. The identification of coarse dietary categories using 2D methods (at 100x magnification) may still be possible; however, caution should be taken in comparing data between different observers.

## Materials and Methods

### Materials

Carnivorans included in this study consist of the following extant species: *Acinonyx* *jubatus* (cheetah, *n*=9), *Panthera* *leo* (African lion, *n*=15), and *Crocuta* *crocuta* (spotted hyena, *n*=12). All extant carnivoran data including descriptive statistics are reported in Ref. [23]. Bovids included in this study are from Scott [21] and consist of the following extant species: *Antidorcas* *marsupialis* (springbok, *n*=10), *Cephalophus* *sylvicultor* (yellow-backed duiker, *n*=10), *Damiliscus* *lunatus* (common tsessebe, *n*=10), and *Sylvicapra* *grimmia* (common duiker, *n*=10). All specimens examined in this study are housed in publicly accessible collections and were examined and molded while visiting respective museums (permission to mold and study all included specimens was granted by all listed museums), including the American Museum of Natural History (AMNH), Field Museum of Natural History (FMNH), National Museum of Natural History (NMNH), Iziko South African Museum (SAM), and, Royal Museum for Central Africa (RMCA). All dental microwear texture data were previously analyzed and published [21,23]; however, the photosimulations of prior analyses are here measured for 2D dental microwear features.

### Dental Microwear

The enamel region of the lower carnassial shearing facet of the M_1_ trigonid was examined on all carnivoran specimens [23], and on all bovid specimens the disto-buccal enamel band of the mesial cuspoid of the M_2_ was examined [21]. All analyzed facets were first cleaned with cotton swabs soaked in acetone to remove preservative (e.g., Butvar). Once the tooth was dry, a mold was made using polyvinylsiloxane dental impression material (President’s Jet regular body, Coltène-Whaledent Corp., Cuyahoga Falls, OH, USA). Tooth casts were then prepared using Epotek 301 epoxy resin and hardener (Epoxy Technologies Corp., Billerica, MA, USA).

Dental microwear texture analysis (DMTA) was performed on all casts that preserved ante mortem microwear using white-light confocal profilometry and scale-sensitive fractal analysis (SSFA) [18–23]. All specimens were scanned in three dimensions in four adjacent fields of view at 100x magnification, for a total sampled area of 204 x 276 µm^2^. These scans, labeled a-d, were converted to photosimulation bitmap images for 2D dental microwear analysis via Solarmap Universal software (Solarius Development, Inc.). At the magnification and total sampling area mentioned above, features ≥2 µm are likely to be well resolved. All bitmaps were renamed with a randomly assigned number and made accessible to all observers for counting. Four observers counted each a-d scan of all taxa per dietary group, for a total of 144 carnivoran and 160 bovid scans. None of the observers were aware of what taxa were being studied or the identification of individual scans, when collecting data. All photosimulations were saved at 28.35 dots per cm (dpcm) and viewed on computer monitor screens with resolutions of ≥ 35.43 dpcm. Observers counted the number of pits, coarse pits, scratches, and coarse scratches in each bitmap (as defined by [11]), with microwear index calculated from scratch and pit counts (number of scratches/number of pits) [36]. As Ref. [11] is the most commonly employed method by those primarily studying non-primate herbivorous and carnivorous mammals, we have quantified 2D microwear using this method (in contrast to other similar microwear methods, including those that use imaging or microwear software). Median dental microwear variables were calculated per specimen per observer (as is done with DMTA attributes) and subsequently analyzed, except when explicitly noted. All image files are available upon request.

Tooth surfaces were analyzed for DMTA attributes including: complexity, scale of maximum complexity, anisotropy, heterogeneity, and textural fill volume. Complexity (*Asfc*) distinguishes taxa that consume brittle foods from taxa that consume softer and/or tougher ones, by assessing the change in surface roughness across changing scale of observation [19,20]. Organisms that consume harder and/or more brittle food items have higher complexity [19–25]. Scale of maximum complexity (Smc) measures the fine-scale limits of the *Asfc* with greater *Smc* surface values associated with fewer small features [20]. Anisotropy (*epLsar*) represents the degree that features of similar depth share a similar orientation (e.g., lots of parallel striations yield more anisotropic surfaces) [19,20]. Grazers or flesh consumers typically have higher anisotropy due to the presence of many parallel scratches of similar depth [19–25]. Heterogeneity (*HAsfc*_(3x3)_ and *HAsfc*_(9x9)_), the degree of texture complexity variation, is measured by calculating *Asfc* variation among subdivided samples (a 3x3 and 9x9 grid, totaling 9 to 81 subsamples, respectively) [19,20]. Thus, surfaces with high heterogeneity have greater disparity in complexity values between subdivided samples and the entire surface. Lastly, textural fill volume (Tfv) measures the volume filled by large (10 µm diameter) and small (2 µm diameter) square cuboids, with high *Tfy* values indicating potentially deeper and/or larger features [20–25]. Scale sensitive fractal analysis software (ToothFrax and SFrax, Surfract Corp., www.surfrait.com) was used to analyze all scans and characterize tooth surfaces.

### Statistical Analysis

Normality tests (Shapiro-Wilk tests, Tables 1, 2) were run on all 2D and 3D variables. However, as the majority of dental microwear variables are not normally distributed we used non-parametric statistical tests (Kruskal-Wallis) to compare differences among all taxa and between observers, except when noted. Carnivorous and herbivorous mammals were analyzed separately. We used Dunn’s procedure [37] and equivalent parametric tests (i.e., Fisher LSD and Tukey HSD tests) when appropriate to conduct multiple comparisons between taxa within like dietary groups (i.e., herbivores-carnivores). Additionally, we compared the two most experienced microwear counters per dietary group, using paired Wilcoxon signed-rank tests and Student’s t-tests (when normally distributed). When comparing observer differences, median microwear values were compared between different individuals (however, comparisons of all individual scans were also made and noted in the results and Table S5). We calculated interobserver error based on Ref. [4] using 2D microwear attributes for all photosimulations analyzed per dietary group (i.e., 144 in carnivorans, 160 in bovids), including all four observers and the two most trained observers. Note, when comparing only two observers, interobserver error can never exceed 100%, mathematically. Correlations between DMTA feature attributes and traditional dental microwear characters (e.g., pit and scratch frequency) were analyzed using Spearman’s rank correlation coefficients (ρ).

## Supporting Information

Appendix S1 All observer counts for all scans of all extant taxa included in this study.
(XLS)

Table S1 All carnivoran specimens examined and 3D dental microwear texture attributes.
(DOC)

Table S2 All bovid specimens examined and 3D dental microwear texture attributes.
(DOC)

Table S3 All carnivoran specimens examined and 2D dental microwear character averages between four observers.
(DOC)

Table S4 All bovid specimens examined and 2D dental microwear character averages between four observers.
(DOC)

Table S5 Summary of comparisons using Dunn’s procedure of observer differences between all individual scans of all extant carnivorans analyzed.
(DOC)
