# Supplementary material for: Direct Comparisons of 2D and 3D Dental Microwear Proxies in Extant Herbivorous and Carnivorous Mammals
Source: PLoS One. 2013 Aug 6;8(8):e71428. doi: 10.1371/journal.pone.0071428 (PMC3735535; doi:10.1371/journal.pone.0071428)
Supplement: Table S1 — All carnivoran specimens examined and 3D dental microwear texture attributes. (DOC) [file pone.0071428.s002.doc]

**Table S1.** All carnivoran specimens examined and 3D dental microwear texture attributes.

| Taxon | Diet | Museum | ID | *Asfc* | *epLsar* | *Smc* | *Tfv* | *HAsfc*_(3x3)_ | *HAsfc*_(9x9)_ |
| --- | --- | --- | --- | --- | --- | --- | --- | --- | --- |
| *Acinonyx jubatus* | no durophagy | AMNH | 27897 | 1.821 | 0.0064 | 0.151 | 8347 | 0.952 | 1.698 |
|  |  | AMNH | 119654 | 0.942 | 0.0045 | 0.341 | 11050 | 0.512 | 1.032 |
|  |  | AMNH | 119656 | 0.817 | 0.0064 | 0.418 | 1618 | 0.399 | 0.767 |
|  |  | AMNH | 119657 | 1.111 | 0.0045 | 0.209 | 38 | 0.543 | 2.237 |
|  |  | AMNH | 161139 | 0.759 | 0.0038 | 0.150 | 0 | 0.739 | 1.070 |
|  |  | SAM | 36849 | 2.645 | 0.0047 | 0.152 | 534 | 1.090 | 3.257 |
|  |  | SAM | 38624 | 2.674 | 0.0056 | 0.208 | 6833 | 0.373 | 0.734 |
|  |  | USNM | 161922 | 1.767 | 0.0030 | 0.601 | 2581 | 0.345 | 0.799 |
|  |  | USNM | 540001 | 1.772 | 0.0051 | 0.345 | 14638 | 0.344 | 0.539 |
| *Crocuta crocuta* | high degree of durophagy | AMNH | 20809 | 6.867 | 0.0038 | 0.150 | 16323 | 0.372 | 0.627 |
|  |  | AMNH | 83591 | 2.280 | 0.0027 | 0.151 | 1234 | 0.604 | 1.314 |
|  |  | AMNH | 83592 | 23.864 | 0.0019 | 0.152 | 15255 | 0.384 | 0.672 |
|  |  | AMNH | 187771 | 16.047 | 0.0052 | 0.150 | 19699 | 0.250 | 0.581 |
|  |  | AMNH | 187772 | 4.988 | 0.0036 | 0.151 | 10135 | 0.320 | 0.556 |
|  |  | AMNH | 187774 | 7.273 | 0.0026 | 0.151 | 11482 | 0.532 | 1.014 |
|  |  | SAM | 33341 | 5.669 | 0.0023 | 0.150 | 13029 | 0.790 | 1.435 |
|  |  | SAM | 33432 | 4.594 | 0.0036 | 0.151 | 7761 | 0.507 | 0.829 |
|  |  | SAM | 36871 | 10.538 | 0.0032 | 0.150 | 15994 | 0.262 | 0.458 |
|  |  | SAM | 40361 | 18.553 | 0.0037 | 0.150 | 17531 | 0.446 | 0.575 |
|  |  | SAM | 83593 | 7.658 | 0.0012 | 0.151 | 15610 | 0.328 | 0.729 |
|  |  | SAM | 38817b | 3.445 | 0.0036 | 0.151 | 3783 | 0.752 | 1.243 |
| *Panthera leo* | intermediate degree of durophagy | AMNH | 17274 | 4.690 | 0.0033 | 0.150 | 7463 | 0.416 | 0.784 |
|  |  | AMNH | 39870 | 4.792 | 0.0029 | 0.150 | 12095 | 0.638 | 1.068 |
|  |  | AMNH | 52072 | 7.354 | 0.0041 | 0.150 | 8860 | 0.513 | 0.826 |
|  |  | AMNH | 81830 | 4.723 | 0.0009 | 0.151 | 10966 | 0.633 | 1.706 |
|  |  | AMNH | 81836 | 6.227 | 0.0019 | 0.151 | 13425 | 0.535 | 0.829 |
|  |  | SAM | 3983 | 5.929 | 0.0022 | 0.153 | 11358 | 0.831 | 1.357 |
|  |  | SAM | 14893 | 1.807 | 0.0075 | 2.418 | 14934 | 0.417 | 0.693 |
|  |  | SAM | 36873 | 6.050 | 0.0046 | 0.150 | 14769 | 0.511 | 1.158 |
|  |  | SAM | 36874 | 4.617 | 0.0034 | 0.150 | 3852 | 0.541 | 0.951 |
|  |  | SAM | 38222 | 7.132 | 0.0034 | 0.151 | 14318 | 0.309 | 0.799 |
|  |  | SAM | 39302 | 2.110 | 0.0011 | 0.816 | 10307 | 0.442 | 0.783 |
|  |  | USNM | 182297 | 2.582 | 0.0017 | 0.150 | 3710 | 0.263 | 0.535 |
|  |  | USNM | 216602 | 4.487 | 0.0017 | 0.150 | 3774 | 0.266 | 0.598 |
|  |  | USNM | 236919 | 3.075 | 0.0045 | 0.150 | 11433 | 0.427 | 0.630 |
|  |  | USNM | 236920 | 3.665 | 0.0041 | 10.147 | 14929 | 0.320 | 0.701 |

*Asfc*, area-scale fractal complexity; *epLsar*, anisotropy; *Smc*, scale of maximum complexity; *Tfv*, textural fill volume; *HAsfc*_(3x3)_, *HAsfc*_(9x9)_ heterogeneity of complexity in a 3x3 and 9x9 grid, respectively. All DMTA data were previously published in Ref. 23.
