# Supplementary material for: Direct Comparisons of 2D and 3D Dental Microwear Proxies in Extant Herbivorous and Carnivorous Mammals
Source: PLoS One. 2013 Aug 6;8(8):e71428. doi: 10.1371/journal.pone.0071428 (PMC3735535; doi:10.1371/journal.pone.0071428)
Supplement: Table S2 — All bovid specimens examined and 3D dental microwear texture attributes. (DOC) [file pone.0071428.s003.doc]

**Table S2.** All bovid specimens examined and 3D dental microwear texture attributes.

*Asfc*, area-scale fractal complexity; *epLsar*, anisotropy; *Smc*, scale of maximum complexity; *Tfv*, textural fill volume; *HAsfc*_(3x3)_, *HAsfc*_(9x9)_ heterogeneity of complexity in a 3x3 and 9x9 grid, respectively. All DMTA data were previously published in Ref. 21.

| Taxon | Diet | Museum | ID | *Asfc* | *epLsar* | *Smc* | *Tfv* | *HAsfc*_(3x3)_ | *HAsfc*_(9x9)_ |
| --- | --- | --- | --- | --- | --- | --- | --- | --- | --- |
| *Antidorcas marsupialis* | browser-grazer | FMNH | 129730 | 1.794 | 0.0036 | 0.268 | 4839 | 0.437 | 0.694 |
|  |  | FMNH | 140888 | 1.570 | 0.0038 | 0.267 | 8428 | 0.518 | 0.906 |
|  |  | FMNH | 31254 | 1.285 | 0.0024 | 0.342 | 4429 | 0.606 | 0.820 |
|  |  | FMNH | 34490 | 2.771 | 0.0044 | 0.816 | 6470 | 0.346 | 0.522 |
|  |  | FMNH | 34493 | 1.658 | 0.0020 | 0.268 | 5561 | 0.646 | 0.893 |
|  |  | FMNH | 34510 | 1.677 | 0.0038 | 0.435 | 5376 | 0.583 | 0.982 |
|  |  | FMNH | 52061 | 1.694 | 0.0056 | 0.508 | 5390 | 0.465 | 0.571 |
|  |  | FMNH | 52143 | 2.064 | 0.0031 | 0.150 | 6286 | 0.407 | 0.700 |
|  |  | FMNH | 75351 | 2.292 | 0.0057 | 0.266 | 4391 | 0.850 | 0.799 |
|  |  | FMNH | 85999 | 2.566 | 0.0029 | 0.342 | 5923 | 0.410 | 0.800 |
| *Cephalophus sylvicultor* | frugivore | RMCA | 83-006M483 | 3.103 | 0.0013 | 0.269 | 10327 | 0.565 | 0.872 |
|  |  | RMCA | 83-006M484 | 5.399 | 0.0040 | 0.268 | 13814 | 0.415 | 0.858 |
|  |  | RMCA | 83-006M485 | 6.729 | 0.0021 | 0.258 | 14438 | 0.519 | 0.762 |
|  |  | RMCA | 83-006M486 | 3.643 | 0.0036 | 0.264 | 13247 | 0.465 | 0.873 |
|  |  | RMCA | 83-006M489 | 4.448 | 0.0019 | 0.233 | 13528 | 0.428 | 0.858 |
|  |  | RMCA | 83-006M493 | 6.921 | 0.0016 | 0.225 | 14917 | 0.497 | 0.831 |
|  |  | RMCA | 83-006M495 | 4.793 | 0.0041 | 0.285 | 14862 | 0.494 | 0.692 |
|  |  | RMCA | 83-006M498 | 4.628 | 0.0018 | 0.208 | 9497 | 0.426 | 0.816 |
|  |  | RMCA | 83-006M499 | 6.658 | 0.0030 | 0.294 | 14279 | 0.427 | 0.812 |
|  |  | RMCA | 93-006M487 | 3.081 | 0.0036 | 0.267 | 11061 | 0.427 | 0.824 |
| *Damaliscus lunatus* | grazer | FMNH | 104429 | 0.645 | 0.0077 | 0.908 | 1998 | 0.458 | 0.640 |
|  |  | FMNH | 127990 | 0.845 | 0.0086 | 1.066 | 1547 | 0.387 | 0.556 |
|  |  | FMNH | 135326 | 1.347 | 0.0062 | 1.434 | 1249 | 0.488 | 0.748 |
|  |  | FMNH | 161157 | 0.934 | 0.0060 | 1.150 | 1270 | 0.477 | 0.673 |
|  |  | FMNH | 19595 | 0.761 | 0.0066 | 1.433 | 3603 | 0.562 | 0.692 |
|  |  | FMNH | 19596 | 1.275 | 0.0071 | 1.343 | 1327 | 0.444 | 0.609 |
|  |  | FMNH | 27481 | 1.039 | 0.0074 | 2.141 | 2771 | 0.523 | 0.767 |
|  |  | FMNH | 29529 | 0.734 | 0.0059 | 1.072 | 3674 | 0.395 | 0.547 |
|  |  | FMNH | 29531 | 1.350 | 0.0061 | 1.733 | 1049 | 0.396 | 0.674 |
|  |  | FMNH | 34527 | 0.909 | 0.0065 | 1.267 | 1786 | 0.314 | 0.671 |
| *Sylvicapra grimmia* | browser | FMNH | 17789 | 3.612 | 0.0014 | 1.141 | 9429 | 0.608 | 1.266 |
|  |  | FMNH | 17790 | 3.343 | 0.0021 | 0.501 | 16032 | 0.930 | 0.906 |
|  |  | FMNH | 28489 | 1.617 | 0.0033 | 0.383 | 15816 | 0.615 | 1.049 |
|  |  | FMNH | 8166 | 2.045 | 0.0020 | 0.509 | 8258 | 0.467 | 0.858 |
|  |  | FMNH | 84006 | 3.933 | 0.0017 | 1.109 | 10112 | 0.556 | 0.713 |
|  |  | FMNH | unid10 | 3.613 | 0.0028 | 1.200 | 15308 | 0.444 | 0.760 |
|  |  | RMCA | 14838 | 4.175 | 0.0037 | 0.509 | 11599 | 1.303 | 0.913 |
|  |  | RMCA | 21794 | 4.508 | 0.0032 | 0.435 | 14950 | 1.324 | 0.732 |
|  |  | RMCA | 35109 | 3.063 | 0.0037 | 0.511 | 11621 | 0.435 | 0.718 |
|  |  | RMCA | 7911 | 3.271 | 0.0027 | 0.510 | 13093 | 1.099 | 1.040 |
