# Supplementary material for: Direct Comparisons of 2D and 3D Dental Microwear Proxies in Extant Herbivorous and Carnivorous Mammals
Source: PLoS One. 2013 Aug 6;8(8):e71428. doi: 10.1371/journal.pone.0071428 (PMC3735535; doi:10.1371/journal.pone.0071428)
Supplement: Table S3 — All carnivoran specimens examined and 2D dental microwear character averages between four observers. (DOC) [file pone.0071428.s004.doc]

**Table S3.** All carnivoran specimens examined and 2D dental microwear feature averages between four observers.

| Taxon | Diet | Museum | ID | Pits | Coarse Pits | Scratches | Coarse Scratches | Microwear Index |
| --- | --- | --- | --- | --- | --- | --- | --- | --- |
| *Acinonyx jubatus* | no durophagy | AMNH | 27897 | 7.3 | 1.0 | 30.8 | 2.8 | 2.51 |
|  |  | AMNH | 119654 | 30.8 | 1.8 | 20.3 | 2.3 | 0.61 |
|  |  | AMNH | 119656 | 21.0 | 2.3 | 5.5 | 0.8 | 0.21 |
|  |  | AMNH | 119657 | 17.3 | 1.3 | 16.8 | 0.8 | 0.66 |
|  |  | AMNH | 161139 | 18.3 | 3.3 | 19.8 | 2.3 | 0.77 |
|  |  | SAM | 36849 | 21.5 | 2.5 | 17.0 | 1.0 | 0.57 |
|  |  | SAM | 38624 | 19.8 | 1.8 | 23.0 | 1.5 | 0.98 |
|  |  | USNM | 161922 | 17.3 | 3.5 | 9.5 | 1.3 | 0.34 |
|  |  | USNM | 540001 | 28.5 | 2.3 | 13.8 | 1.5 | 0.40 |
| *Crocuta crocuta* | high degree of durophagy | AMNH | 20809 | 29.3 | 3.0 | 8.5 | 0.0 | 0.32 |
|  |  | AMNH | 83591 | 23.3 | 3.0 | 6.0 | 0.0 | 0.26 |
|  |  | AMNH | 83592 | 25.3 | 3.5 | 6.0 | 0.0 | 0.26 |
|  |  | AMNH | 187771 | 23.3 | 3.5 | 12.3 | 0.8 | 0.43 |
|  |  | AMNH | 187772 | 21.8 | 2.5 | 20.5 | 1.3 | 0.77 |
|  |  | AMNH | 187774 | 14.8 | 3.0 | 5.8 | 0.0 | 0.29 |
|  |  | SAM | 33341 | 22.5 | 4.0 | 6.3 | 0.3 | 0.26 |
|  |  | SAM | 33432 | 20.3 | 3.0 | 14.0 | 0.3 | 0.54 |
|  |  | SAM | 36871 | 30.8 | 2.8 | 8.3 | 1.3 | 0.30 |
|  |  | SAM | 40361 | 25.5 | 3.5 | 13.8 | 0.8 | 0.52 |
|  |  | SAM | 83593 | 15.5 | 3.5 | 1.3 | 0.0 | 0.08 |
|  |  | SAM | 38817b | 20.5 | 4.0 | 5.3 | 0.0 | 0.22 |
| *Panthera leo* | intermediate degree of durophagy | AMNH | 17274 | 29.5 | 4.0 | 9.0 | 1.8 | 0.33 |
|  |  | AMNH | 39870 | 23.3 | 4.3 | 17.5 | 0.8 | 0.58 |
|  |  | AMNH | 52072 | 18.8 | 3.0 | 7.5 | 1.0 | 0.30 |
|  |  | AMNH | 81830 | 27.8 | 3.5 | 11.8 | 0.8 | 0.43 |
|  |  | AMNH | 81836 | 44.8 | 3.3 | 16.5 | 2.0 | 0.43 |
|  |  | SAM | 3983 | 20.3 | 2.3 | 8.0 | 1.3 | 0.34 |
|  |  | SAM | 14893 | 28.0 | 2.5 | 6.0 | 0.0 | 0.22 |
|  |  | SAM | 36873 | 27.0 | 3.8 | 21.3 | 1.5 | 0.75 |
|  |  | SAM | 36874 | 34.8 | 4.0 | 4.8 | 0.3 | 0.18 |
|  |  | SAM | 38222 | 21.8 | 3.3 | 7.0 | 0.3 | 0.27 |
|  |  | SAM | 39302 | 34.0 | 4.5 | 8.3 | 0.8 | 0.24 |
|  |  | USNM | 182297 | 29.5 | 3.3 | 7.3 | 0.8 | 0.26 |
|  |  | USNM | 216602 | 37.5 | 3.8 | 16.0 | 1.0 | 0.42 |
|  |  | USNM | 236919 | 20.5 | 1.3 | 23.3 | 2.3 | 1.04 |
|  |  | USNM | 236920 | 23.8 | 2.8 | 9.0 | 0.3 | 0.41 |

Number of pits, coarse pits, scratches, and coarse scratches, defined by Ref. 11. Microwear index, number of scratches/number of pits (Ref. 36). All 2D dental microwear features are averages of median values taken from four photosimulations per specimen (see Materials and Methods).
