# Supplementary material for: Direct Comparisons of 2D and 3D Dental Microwear Proxies in Extant Herbivorous and Carnivorous Mammals
Source: PLoS One. 2013 Aug 6;8(8):e71428. doi: 10.1371/journal.pone.0071428 (PMC3735535; doi:10.1371/journal.pone.0071428)
Supplement: Table S4 — All bovid specimens examined and 2D dental microwear character averages between four observers. (DOC) [file pone.0071428.s005.doc]

**Table S4.** All bovid specimens examined and 2D dental microwear feature averages between four observers.

| Taxon | Diet | Museum | ID | Pits | Coarse Pits | Scratches | Coarse Scratches | Microwear Index |
| --- | --- | --- | --- | --- | --- | --- | --- | --- |
| *Antidorcas marsupialis* | browser-grazer | FMNH | 129730 | 32.1 | 4.6 | 12.0 | 1.1 | 0.51 |
|  |  | FMNH | 140888 | 29.4 | 5.8 | 17.3 | 1.3 | 0.68 |
|  |  | FMNH | 31254 | 29.3 | 6.0 | 22.3 | 3.0 | 0.89 |
|  |  | FMNH | 34490 | 26.1 | 2.1 | 16.9 | 1.5 | 1.03 |
|  |  | FMNH | 34493 | 32.5 | 7.0 | 20.6 | 0.8 | 0.72 |
|  |  | FMNH | 34510 | 45.5 | 12.4 | 17.6 | 1.0 | 0.43 |
|  |  | FMNH | 52061 | 39.1 | 7.1 | 7.5 | 0.0 | 0.20 |
|  |  | FMNH | 52143 | 44.0 | 9.1 | 11.3 | 0.5 | 0.28 |
|  |  | FMNH | 75351 | 19.1 | 2.9 | 8.0 | 0.1 | 0.60 |
|  |  | FMNH | 85999 | 40.0 | 11.4 | 10.5 | 0.4 | 0.32 |
| *Cephalophus sylvicultor* | frugivore | RMCA | 83-006M483 | 32.9 | 1.3 | 3.4 | 0.1 | 0.17 |
|  |  | RMCA | 83-006M484 | 46.3 | 6.5 | 3.1 | 0.3 | 0.07 |
|  |  | RMCA | 83-006M485 | 33.4 | 2.8 | 0.8 | 0.3 | 0.02 |
|  |  | RMCA | 83-006M486 | 37.5 | 3.5 | 1.9 | 0.0 | 0.05 |
|  |  | RMCA | 83-006M489 | 35.4 | 2.5 | 1.8 | 0.0 | 0.07 |
|  |  | RMCA | 83-006M493 | 12.8 | 2.0 | 3.6 | 0.0 | 0.30 |
|  |  | RMCA | 83-006M495 | 14.1 | 1.8 | 1.5 | 0.0 | 0.08 |
|  |  | RMCA | 83-006M498 | 36.5 | 5.3 | 1.1 | 0.0 | 0.04 |
|  |  | RMCA | 83-006M499 | 29.4 | 2.3 | 1.6 | 0.1 | 0.07 |
|  |  | RMCA | 93-006M487 | 40.8 | 3.1 | 1.8 | 0.4 | 0.04 |
| *Damaliscus lunatus* | grazer | FMNH | 104429 | 25.8 | 2.3 | 19.4 | 2.9 | 1.93 |
|  |  | FMNH | 127990 | 53.8 | 2.8 | 28.9 | 2.4 | 1.09 |
|  |  | FMNH | 135326 | 26.8 | 4.4 | 18.0 | 1.8 | 0.79 |
|  |  | FMNH | 161157 | 44.0 | 8.3 | 24.9 | 1.0 | 0.68 |
|  |  | FMNH | 19595 | 58.4 | 4.0 | 27.9 | 2.6 | 0.84 |
|  |  | FMNH | 19596 | 58.8 | 1.0 | 25.0 | 2.3 | 1.22 |
|  |  | FMNH | 27481 | 47.1 | 3.3 | 16.3 | 2.1 | 0.66 |
|  |  | FMNH | 29529 | 35.9 | 5.4 | 23.9 | 1.4 | 0.89 |
|  |  | FMNH | 29531 | 18.1 | 2.4 | 19.6 | 3.5 | 1.84 |
|  |  | FMNH | 34527 | 22.6 | 2.5 | 16.6 | 1.0 | 0.95 |
| *Sylvicapra grimmia* | browser | FMNH | 17789 | 22.3 | 3.3 | 24.9 | 4.5 | 1.50 |
|  |  | FMNH | 17790 | 21.5 | 2.8 | 21.1 | 1.6 | 1.26 |
|  |  | FMNH | 28489 | 20.0 | 3.0 | 19.8 | 2.1 | 1.19 |
|  |  | FMNH | 8166 | 24.1 | 4.6 | 18.5 | 2.9 | 0.91 |
|  |  | FMNH | 84006 | 22.8 | 3.4 | 14.4 | 0.5 | 0.80 |
|  |  | FMNH | unid10 | 36.9 | 5.0 | 25.1 | 2.0 | 0.93 |
|  |  | RMCA | 14838 | 23.4 | 2.8 | 20.4 | 4.5 | 1.37 |
|  |  | RMCA | 21794 | 24.5 | 2.9 | 18.6 | 3.8 | 0.94 |
|  |  | RMCA | 35109 | 16.1 | 4.3 | 23.8 | 3.0 | 1.75 |
|  |  | RMCA | 7911 | 28.0 | 4.5 | 15.4 | 0.9 | 0.72 |

Number of pits, coarse pits, scratches, and coarse scratches, defined by Ref 11. Microwear index, number of scratches/number of pits (Ref. 36). All 2D dental microwear features are averages of median values taken from four photosimulations per specimen (see Materials and Methods).
