# Supplementary material for: Direct Comparisons of 2D and 3D Dental Microwear Proxies in Extant Herbivorous and Carnivorous Mammals
Source: PLoS One. 2013 Aug 6;8(8):e71428. doi: 10.1371/journal.pone.0071428 (PMC3735535; doi:10.1371/journal.pone.0071428)
Supplement: Table S5 — Summary of comparisons using Dunn’s procedure of observer differences between all individual scans of all extant carnivorans analyzed. (DOC) [file pone.0071428.s006.doc]

**Table S5.** Summary of comparisons using Dunn’s procedure of observer differences between all individual scans of all extant carnivorans analyzed.

| **Dietary Group** | **Taxon** | ***n*** | **Pits** | **CP** | **Scratches** | **CS** | **MI** |
| --- | --- | --- | --- | --- | --- | --- | --- |
| *carnivore, avoids bone processing* | *Acinonyx jubatus* | 36 | **p<0.0001*** | **p<0.0001*** | p=0.052 | **p<0.0001*** | **p<0.0001*** |
| *carnivore, generalized degree of durophagy* | *Panthera leo* | 60 | **p<0.0001*** | **p<0.0001*** | **p=0.006*** | **p<0.0001*** | **p=0.007*** |
| *carnivore, high degree of durophagy* | *Crocuta crocuta* | 48 | **p<0.0001*** | **p<0.0001*** | **p=0.007*** | **p<0.0001*** | **p<0.001*** |

*Significant values (*p*<0.05). *n,* number of individuals sampled; Pits, number of pits of all size categories; CP, number of coarse pits as defined by Ref. 11; Scratches, number of scratches of all size categories; CS, number of coarse scratches as defined by Ref. 11; MI, microwear index as defined by the number of scratches/number of pits, Ref. 36. All 2D dental microwear features analyzed are from each of four photosimulations per specimen per observer (see Materials and Methods). Note, bovids were not analyzed as statistical comparisons of mean values were already significant (Table 3).
